# Rifaximin as a rare cause of rhabdomyolysis in cirrhosis

**DOI:** 10.1002/ccr3.1583

**Published:** 2018-05-27

**Authors:** Alec B. Rezigh, Erin Armenia, Ting Li, Irene Soesilo

**Affiliations:** ^1^ Internal Medicine Presbyterian St. Lukes Medical Center Denver CO USA; ^2^ Internal Medicine University of Colorado at Denver ‐ Anschutz Medical Campus Aurora CO USA

**Keywords:** adverse medication reaction, cirrhosis, rhabdomyolysis, rifaximin

## Abstract

Rifaximin has only been rarely reported to cause rhabdomyolysis. When physical and nonphysical etiologies have been excluded, thorough review of the patient’s medication list is necessary. In cirrhotics, the potential harm of rifaximin in treatment or prophylaxis of hepatic encephalopathy should be recognized.

## 1 INTRODUCTION

Rhabdomyolysis is the clinical syndrome defined by muscle death and the subsequent release of intracellular components. It is often characterized by a constellation of elevated creatine kinase, muscle pain and/or weakness, and myoglobinuria.^1^ The etiology can often be determined by the history, physical examination, and laboratory findings. Here we report a case of rhabdomyolysis caused by an uncommonly implicated medication.

## 2 CASE

The patient is a 53‐year‐old male with a history of alcoholic cirrhosis, who presented with a one‐day history of severe generalized muscle weakness and myalgias; he was unable to rise from a chair without assistance. His cirrhosis was diagnosed in October 2016 per clinical history and findings on Computed Tomography (CT). His disease was complicated by Grade 2 esophageal varices, ascites, and hepatic encephalopathy (HE). He also had one hospitalization approximately 1 month prior to admission for an upper gastrointestinal bleed (UGIB), requiring four variceal bands. He had no history of spontaneous bacterial peritonitis (SBP). On admission, his model for end‐stage liver disease (MELD) score was 20; his Child‐Pugh Score was 12, placing him in Class C. His last drink was 32 days prior to presentation.

On arrival he was afebrile, tachycardic, and mildly hypertensive, but appeared comfortable. Physical examination was remarkable for symmetrical proximal muscle tenderness and weakness ‐ ⅘ in the upper extremities and ⅗ in the lower extremities. Reflexes were 1+ and symmetric; sensation was intact. Laboratory testing was significant for leukocytosis, as well as elevations in the creatinine, aspartate aminotransferase (AST), white blood cells (WBC), and creatine kinase (CK) (Table 1). The patient was also found to have an elevated erythrocyte sedimentation rate (ESR) at 71 mm/h, C‐reactive Protein‐Quantitative (CRP‐QT) at 2.7 mg/dL, and lactate 3.5 mmol/L. Chronic abnormalities in his hemoglobin, albumin, INR, bilirubin, and alkaline phosphatase were also present, although remained stable throughout admission. Urinalysis demonstrated large blood on the dipstick, but was likely due to myoglobin, as microscopy revealed only 0‐5 RBCs per high‐power field. The etiology of his rhabdomyolysis was not apparent: he denied seizures, trauma, increased exertion, prolonged immobilization, recent travel, or use of statins, supplements, alcohol, or illicit drugs.

**Table 1:** Laboratory values for Days 1‐12 of admission and Day 91 at an outpatient visit

	CK (units/L)	Cr (mg/dL)	AST (units/L)	WBC (K/mm3)
Day 1	13 306	1.4	721	18.93
Day 2	8630	1.4	516	18.2
Day 3	9150	1.2	566	17.94
Day 4	10 766	1	647	17.98
Day 5	14 833	0.9	822	18.14
Day 6	19 197	1	1080	16.85
Day 7	13 029	1.2	963	17.11
Day 8	9552	0.9	850	15.33
Day 9	6275	0.8	745	14.82
Day 10	5131	0.9	635	14.07
Day 11	2790	0.9	523	12.02
Day 12	1594	0.8	413	13.43
Day 91	152	0.9	47	6.77

**Figure 1:**
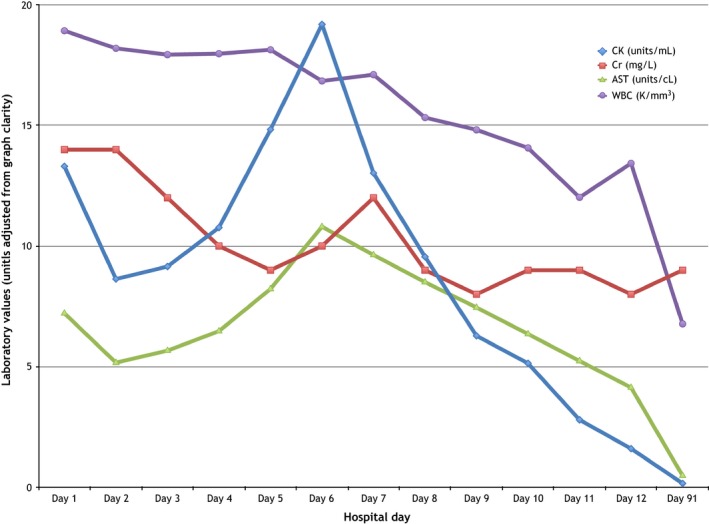
Laboratory trends during the hospital course, with initial improvement and recrudescence of the CK, AST, and WBC above admission values despite normalization of the Cr (baseline 9‐11 mg/L or 0.9‐1.1 mg/dL) by day 4. This is followed by the notable downtrending of the CK, AST and WBC count immediately following discontinuation of rifaximin on Day 6. Normalization of his laboratories is further documented on Day 91 of his clinical course at an outpatient visit. Traditional units of the aforementioned laboratory tests were adjusted for graphical representation (CK converted from u/L to u/mL, Cr from mg/dL to mg/L, AST from u/L to u/cL, WBC units were not altered)

**Figure 2:**
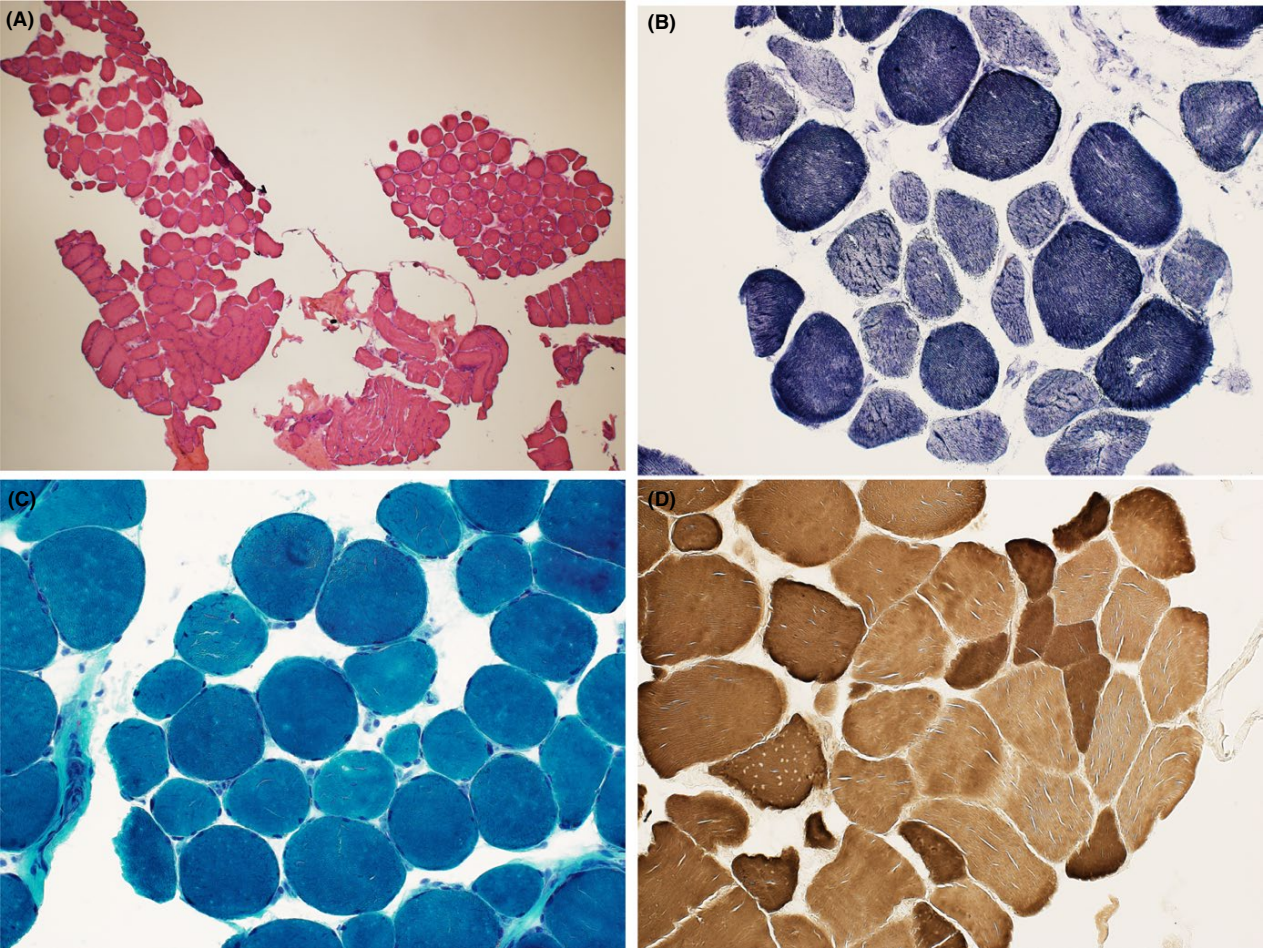
Sample taken from the right thigh. A, Hematoxylin and Eosin stain. B, NADH stain. C, Trichrome stain. D, ATPase stain. Each sample demonstrates slight variation in muscle fiber diameter, which can be explained by mild type‐2 muscle atrophy. This finding is nonspecific and can be present with disuse, exposure to increased steroid levels (which our patient did not receive), cachexia, and disorders of the thyroid. Our patient did meet any of these criteria. The findings were felt to be mild and per the pathologist would not explain his clinical picture of rhabdomyolysis. In images A through D, there is no evidence of inflammation, necrotizing myopathy, vasculitis, or metabolic myopathy to point to an alternate cause of his presentation

He was treated with aggressive intravenous fluid resuscitation. Despite resolution of his acute kidney injury, clearing of his lactate, and initial improvement in his laboratory values, his CK, WBC, and AST plateaued and began to rise again on day four (Figure 1 and Table 1). With ongoing muscle injury and persistent weakness, other etiologies (including autoimmune, infectious, and malignant) were investigated. CT chest/abdomen/pelvis was negative for abscess or mass lesions; muscle biopsy of the right thigh showed no evidence of vasculitis or acquired inflammatory, necrotizing, or metabolic myopathy (Figure 2). Anti‐Jo‐1 and anti‐HMG‐CoA reductase antibodies were both negative. Further medication review revealed he was recently started on rifaximin for hepatic encephalopathy prophylaxis. Rifaximin has been occasionally reported to cause rhabdomyolysis in patients with hepatic insufficiency.^2^ We subsequently held his rifaximin on day six and noted a marked downtrend of CK the next day (Figure 1 and Table 1). The CK continued to downtrend and his myoglobinuria resolved soon after. The patient’s strength continued to improve with therapy, and he was discharged on day 16 with a diagnosis of rifaximin‐induced rhabdomyolysis.

## 3 DISCUSSION

The causes of rhabdomyolysis are numerous and often separated into physical and nonphysical etiologies. Physical etiologies include trauma, arterial occlusion, overexertion, seizures, delirium tremens, electrical injury, and hyperthermia. Nonphysical triggers include metabolic syndromes, toxins/drugs, infections, electrolyte abnormalities, endocrinopathies, and autoimmune conditions.^3^ His history did not reveal any physical triggers. Given his age and absence of family history, metabolic disorders were unlikely. Laboratory work‐up for electrolyte, endocrine, and autoimmune etiologies was negative. Although the patient did have persistent leukocytosis, we felt infectious etiologies were less likely given negative paracentesis, urinalysis, chest X‐ray, respiratory viral panel, and blood cultures. We thus attributed his elevated WBC to reactivity in the setting of inflammation and muscle breakdown. Besides marijuana, he denied exposure to other drugs or toxins. His home medications included gabapentin 300 mg 3 times a day, amlodipine 10 mg daily, cyclobenzaprine 5 mg daily, ranitidine 150 mg daily, and furosemide 20 mg daily. The patient was also taking lactulose 20 g twice a day and rifaximin 550 mg twice a day, started approximately 1 month prior to admission for secondary prevention of HE. This was performed after the patient developed his first and only episode of HE while admitted for an UGIB. Although statin myopathy was considered, our patient had discontinued his atorvastatin 20 mg nightly at the same time his lactulose and rifaximin were initiated. The documented rationale for discontinuation, per review of the records, was to avoid all potentially hepatotoxic agents. Additionally, his muscle biopsy did not reveal signs of necrotizing myopathy, usually seen with statin‐induced injury^4^ (Figure 2). With his biopsy result, negative HMG‐CoA reductase antibody, and history of medication abstinence, statin myopathy was deemed less likely.

Our patient’s unrevealing history and improvement in CK upon drug discontinuation pointed to rifaximin as the etiology of his rhabdomyolysis. Review of the literature revealed one case of rhabdomyolysis with concurrent use of rifaximin and a statin ‐ a nonalcoholic cirrhotic patient on simvastatin for 10 years, who developed rhabdomyolysis 2 weeks after starting rifaximin. Mechanistically, the authors hypothesized rifaximin and its increased absorption in hepatic impairment led to mitochondrial oxidative stress, causing accelerated muscle breakdown.^2^ In patients with normal hepatic function, rifaximin has limited systemic absorption. However, in patients with hepatic impairment (especially patients with Child‐Pugh C or MELD >25), systemic absorption increases.^5^ The reasons behind this are multifactorial, including increased intestinal permeability due to alterations in the mucosal barrier, inflammation, and changes in the gut microbiota.^6^ Cirrhosis also alters the functional ability of p‐glycoprotein, an important efflux pump that transports drugs and toxins out of cells and into the GI lumen, renal tubule, and bile duct.^7^ Further caution must also be taken with concomitant use of p‐glycoprotein inhibitors, such as statins and spironolactone, medications commonly prescribed to cirrhotic patients.^8^ Per review of the FDA MedWatch Reporting system from 1977 to 2017, four people taking rifaximin reported rhabdomyolysis as a side effect.^9^ However, interpretation was limited by the lack of access to their medical records. Although rifaximin was discontinued, it did not appear to be critical to the care of the patient. He was mentating appropriately solely with lactulose and abstinence from EtOH. He had no recurrence of HE and we thus did not seek additional alternative treatments.

In conclusion, rifaximin is a rare, but notable cause of rhabdomyolysis, especially in patients with hepatic insufficiency. As with many clinical presentations, a thorough medication history is paramount when the etiology of a syndrome remains uncertain. If the work‐up remains negative, discontinuation of potential offending agents, even if uncommon, is the next appropriate and important management decision. Providers should recognize the rare, but potential harm of rifaximin use in cirrhotics for the treatment or prophylaxis against hepatic encephalopathy.

## AUTHORSHIP

ABR: collected information on the patient, drafted the manuscript, and approved the final version of the manuscript. EA, TL, and IS: helped in drafting the manuscript and revised contents of the case and discussion of the manuscript.

## CONFLICT OF INTEREST

None declared.
